# WeReview: CRISPR Tools—Live Repository of Computational Tools for Assisting CRISPR/Cas Experiments

**DOI:** 10.3390/bioengineering6030063

**Published:** 2019-07-25

**Authors:** Rafael Torres-Perez, Juan A. Garcia-Martin, Lluis Montoliu, Juan C. Oliveros, Florencio Pazos

**Affiliations:** 1 National Centre for Biotechnology (CNB-CSIC). c/ Darwin, 3. 28049 Madrid, Spain; 2 Rare Diseases Networking Biomedical Research Centre (CIBERER-ISCIII), 28049 Madrid, Spain

**Keywords:** CRISPR/Cas, genome editing, computational tool

## Abstract

Computational tools are essential in the process of designing a CRISPR/Cas experiment for the targeted modification of an organism’s genome. Among other functionalities, these tools facilitate the design of a guide-RNA (gRNA) for a given nuclease that maximizes its binding to the intended genomic site, while avoiding binding to undesired sites with similar sequences in the genome of the organism of interest (off-targets). Due to the popularity of this methodology and the rapid pace at which it evolves and changes, new computational tools show up constantly. This rapid turnover, together with the intrinsic high death-rate of bioinformatics tools, mean that many of the published tools become unavailable at some point. Consequently, the traditional ways to inform the community about the landscape of available tools, i.e., reviews in the scientific literature, are not adequate for this fast-moving field. To overcome these limitations, we have developed “WeReview: CRISPR Tools,” a live, on-line, user-updatable repository of computational tools to assist researchers in designing CRISPR/Cas experiments. In its web site users can find an updated comprehensive list of tools and search for those fulfilling their specific needs, as well as proposing modifications to the data associated with the tools or the incorporation of new ones.

## 1. Introduction

Changing the genomic sequence of a generic organism in a precise, accurate and effective way has been one of the main goals of molecular biology. Such modifications are at the basis of many experimental approaches in this area, and have been shown to be of enormous utility for biotechnological and biomedical applications [1]. There is a long history of methodologies for accomplishing that goal, which present different drawbacks in terms of accuracy, specificity and side effects. Generally, these approaches either were not specific enough (modifying other parts of the genome apart from the targeted ones), were taxon-specific (working only in a particular organisms or clade) or left “residues” in the targeted genome (e.g., footprints and/or genes used during the selection process).

The latest addition to this family of approaches is the Clustered Regularly Interspaced Short Palindromic Repeats/CRISPR-associated (CRISPR/Cas), which enables targeted genome editing in multiple organisms with unprecedented accuracy and specificity [2]. This technique was derived from an ancient and conserved prokaryotic defensive system against foreign DNA [3] and its core is an RNA-dependent DNA nuclease that can be targeted to a desired genomic location with single-nucleotide precision. That genomic site is dictated by the complementarity with the sequence of the guide RNA (gRNA) accompanying the nuclease. Consequently, this nuclease can be “programmed” to act in different parts of the genome by changing the sequence of its gRNA. gRNAs have at their 3’ or 5’ end a fixed sequence required for nuclease recognition, followed by a sequence of 18–25 nucleotides (depending on the nuclease) complementary to the target DNA. An additional constraint when designing the gRNA is that the target genomic sequence must have a short (3–4 nt) motif termed “protospacer adjacent motif” (PAM) in one of its ends that is recognized by the nuclease. The most widely used nuclease in these genome editing experiments is Cas9 from *Streptococcus pyogenes*, but other nucleases are being also used, including Cpf1 [4], SaCas9 [5] and others from different CRISPR/Cas Class II systems [6].

Once the DNA is cleaved by the nuclease at the intended location (dictated by the gRNA), the intrinsic DNA-repairing mechanisms of the organism restore the continuity of the chromosome, while triggering the desired modification. These modifications are very diverse, leading to an ever-expanding CRISPR-based toolbox. Applications range from changing a single nucleotide to introducing/removing long stretches of DNA, repositioning genome loci in the nucleus [7], or simply “labelling” a region by using inactive (dead) nucleases (e.g., dCas9) fused to reporters [8] or to additional protein domains for altering epigenetic marks [9]. Due to its accuracy, relative simplicity, and the broad range of organisms/systems where it can be applied, this technique has shown to be very powerful in different biotechnological and biomedical applications [10]. With the advent of CRISPR-based genome editing techniques, the generation of animal models can now be accomplished faster than was possible with classical techniques [11], including experiments that could not be easily addressed with previous approaches, such as the functional analysis of the non-coding genome [12].

The design of a CRISPR/Cas experiment and the analysis of its outcome unavoidably require using computational tools at different stages. One of the most critical parts is the design of the sequence of the gRNA that will target the nuclease to the desired location. Besides the specific motifs required for being recognized by the nuclease, this gRNA should have the 18–25 nt sequence complementary to that of the targeted DNA site, so that it hybridizes with high affinity there. It is also important to avoid sequences that could hybridize with other genomic locations (eventually with mismatches) besides that targeted, leading to off-target effects. Off-targets are especially critical for the reliability/specificity of this technique and its safety in therapeutic applications, especially those eventually involving human germ-line modifications [13]. The current consensus is that off-target effects can become a problem when the experiment involves continuous expression of the CRISPR/Cas machinery, such as in vitro plasmid transfection, whereas they are probably not relevant when the CRISPR/Cas system is transiently induced (e.g., in vivo, germline-type modifications [14]). For example, in mice, the presence of unintended off-target modifications is rarely observed [12,14]. Consequently, when designing this gRNA computationally, the two main factors to take into account are its affinity (for the target site) and its specificity (to avoid off-targets). Computational tools are involved not only in the design of the experiment, but in the post-analysis of its outcomes as well. For example, using next generation sequencing data, it is possible to assess the genome-wide effects on gene expression of the introduced modifications, both in terms of on- and off-targets.

There are many web-accessible computational tools and databases that help the researcher at all stages of a CRISPR/Cas workflow (for some recent reviews see, for example, [15,16,17,18]). Since CRISPR/Cas is a very hot research topic right now, the field is moving very fast. For example, new nucleases are described continuously, new setups and applications for the system are proposed, new strategies for designing the gRNAs, etc. This is reflected in the ecosystem of associated computational tools: New tools show up at an increasing pace, and many disappear as they become obsolete or are no longer maintained. While the disappearance of computational tools is a generalized problem in Molecular Biology, with important implications for study reproducibility [19], it is particularly serious in the case of CRISPR/Cas-based works due to their high impact—i.e., a prominent published study can come to be irreproducible de facto if the computational tools used become unavailable.

Due to this rapid evolution of the ecosystem of CRISPR-related web tools, the many published reviews become outdated shortly after publication. Consequently, it is difficult for a researcher to find an updated overview of the tools currently available for designing his/her experiment. The large number of tools also makes it difficult to manually locate the one that best fits the researcher’s specific needs. Although there are generic compilations of computational tools, they are not updated frequently, hence their containing old servers. More importantly, as they are not focused on CRISPR/Cas, they do not contain the specific details required by the user of these approaches. For these reasons, we developed “WeReview: CRISPR Tools”, a live, on-line searchable repository of computational tools for CRISPR/Cas.

## 2. Materials and Methods

That initial list of CRISPR-related tools contained in WeReview was compiled from several recent reviews on the subject (i.e., references [15,16,17,18]) as well as on-line generic repositories of bioinformatics tools such as OMICtools [20], the Wikipedia page of CRISPR/Cas Tools (https://en.wikipedia.org/wiki/CRISPR/Cas_Tools) and the online spreadsheet “CRISPR Software Matchmaker” (http://goo.gl/R0gANl). At the time of writing this manuscript, the system contains information for 83 tools. For each tool, some features were manually introduced into the system, while others are automatically retrieved and updated, such as the citation data.

The web interface for WeReview was developed in JavaScript and PHP. It was tested in Google Chrome and Mozilla Firefox.

## 3. Results

The main goal of WeReview is to serve as an updated, on-line, user-updatable repository of computational tools to assist researches in designing CRISPR/Cas experiments. The web interface of WeReview shows a table with the main features of these tools, so that the user can sort the tool list by any of these features, as well as search for those with particular characteristics. It is also possible to add comments, change the contents of the table and incorporate records for new tools.

The features currently included are:

- “Name”: Name of the tool and link to its web site.
- “Available?”: Indicates whether the tool was available the last time checked. In principle, tools unavailable at a given time are not automatically removed since they can eventually become functional again (i.e., a temporary failure). The most recent date the tool was checked for availability is also shown.
- “Purpose”: Main goal of the tool: “Oligo designer,” “database” (database of pre-designed sets of oligos), “post-analysis” (computational analysis of genomic data associated to a CRISPR/Cas experiment) or “other.”
- “Platform”: The way the user can access the tool: “Web” (web interface), “command-line” (local command-line text-based tool), “desktop” (local desktop application with user graphical interface), “webapp” (web service programmatically accessible through an Application Programming Interface (API)).
- “Off-targets”: This indicates whether the tool is able to locate/score potential off-targets for the gRNA.
- “Score oligos”: It shows whether the tool is able to predict the binding efficiency of the gRNA to the intended target(s) in the genome.
- “Search by”: How the intended genomic target has to be specified as input for the tool; e.g., DNA sequence, gene ID or genomic coordinates.
- “Enzyme”: List of Cas nucleases or other enzymes admitted by the system.
- “PAM”: List of “protospacer adjacent motifs” allowed by the tool to be incorporated in the gRNA being designed.
- “Organisms”: List of organisms whose genomes are incorporated into the tool, so that they can be scanned for possible off-targets, etc.
- “Citations”: Total number of articles citing the tool in PubMed Central. A plot representing the yearly citation profile is also shown, so that temporal trends related to the popularity of the tool can be assessed.
- “Reference”: Bibliographic reference of the tool. It includes a link to the corresponding record in PubMed.
- “Comments”: Free-text area to store generic data that do not fit in any of the other fields. For example, it can be used to mention other system objectives besides those formalized in the “Purpose” column, or to indicate that the system is apparently unavailable forever.

**Figure 1:**
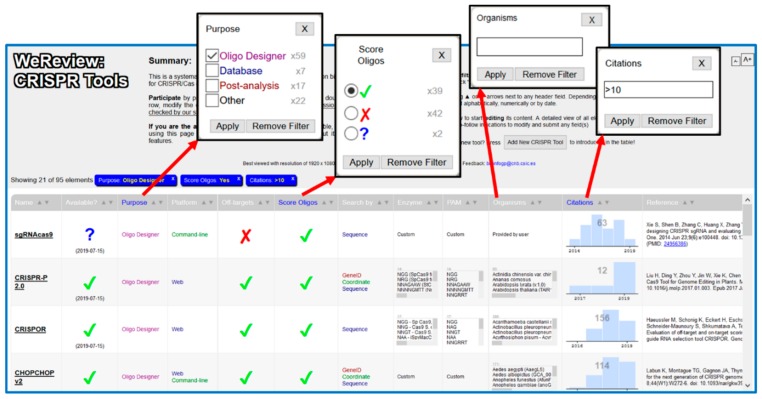
Screenshots of WeReview web interface. The main table, together with some of the dialogs for introducing filters are shown.

It is possible to sort the table by any of these fields by pressing the up/down arrows in the corresponding column. The system also allows filtering the list of tools by searching those fulfilling given criteria. Pressing in the column headers (names of the features), a search box shows up. Depending on the feature, this is either a free-text box or a set of checkboxes (Figure 1). The headers of the columns where filters are being applied are highlighted. Additionally, the list of filters currently being applied is shown above the table (blue boxes). To remove a filter, press the corresponding “X” button on this list.

It is possible to combine many of these searches (filters) to perform complex queries. For example, imagine you are interested in designing a CRISPR/Cas experiment to knock-out a gene in mice using the *S. pyogenes* Cas9 nuclease (PAM: NGG in 3’). Moreover, you want a tool accessible through a web interface and able to calculate the affinity of the gRNA for the target sites. To look for the tools fulfilling these criteria you would select “Web” in “Platform,” the green check in “Score on-targets,” type “NGG” in “PAM” and “mouse” in “Organisms.” With these restrictions you would obtain a list of tools suitable for your experiment. This list could be further filtered or manually inspected, for example to select tools that are available or apparently widely used (i.e., highly cited).

WeReview is intended to be a live tool that can be updated/changed frequently as the CRISPR/Cas field evolves, either by the developers or by the community. To facilitate this, any user can suggest changes to the contents or the addition of a new tool. This is done under a wiki-like schema, without requiring any registration or credentials. Nevertheless, all suggested modifications/additions are moderated, and they have to be approved by the authors of WeReview before being incorporated. For adding a new tool, press the “Add New CRISPR Tool” button in the top right corner and fill the form with the tool data. Some of the fields are free-text while in others the user has to choose among a set of possible values. For the bibliographic reference, just enter the PubMed ID (PMID) and the system will retrieve the corresponding record. Citation data is also automatically retrieved and updated. The user can optionally enter an email address to get feedback from the developers, such as a confirmation if the tool is finally incorporated. To change/edit the data for a given tool, just double-click in the corresponding table row.

“WeReview: CRISPR Tools” is freely available at https://bioinfogp.cnb.csic.es/tools/wereview/crisprtools/.

## 4. Discussion

The motivation behind “WeReview: CRISPR Tools” is to offer interested users the most updated view of the landscape of tools for designing CRISPR/Cas experiments available at any given moment, so that they can explore it interactively and look for the tool that best suits their needs.

Due to the tremendous pace at which the CRISPR/Cas field is evolving, together with the intrinsic high death-rate of bioinformatics tools, it is difficult to have a snapshot of the current landscape of available CRISPR-related computational tools. Even tools that are available and working properly can be “silently” obsolete; for example, if they do not incorporate the latest versions/assemblies of the genomes or those of recently sequenced organisms.

There are many excellent “standard” reviews on the subject which inform the community on the established basis of this technology. Although some include lists of computational tools, these become outdated immediately after publication for the reasons commented. Indeed, in the process of recompiling tools for WeReview and writing this manuscript, some tools became unavailable, while around 30 new ones showed up. 

For the initial set of tools incorporated in WeReview, we tried to be comprehensive, including as many tools as possible, as the user can filter the list according with his/her specific needs. Similarly, for each program we included the fields we considered more useful for the researchers looking for a tool.

While there are many excellent generic repositories of bioinformatics tools, their main drawback regarding the CRISPR/Cas field is that, since they are not specific to it, they do not include critical information required when looking for a tool. For example, while there is a list of CRISPR-related tools in the widely used OMICtools repository [20], it is not possible to filter it by organism or PAM, for example. Consequently, the user would have to go to the original publication of each tool, one by one, to find that information. Moreover, these repositories in general do not allow the community to introduce changes, which can be very important in such a fast-moving field.

Finally, we think that this idea of a “web live list of tools” can be extended many other areas in bioinformatics.
